# Evaluation of magnesium-based scaffolds fabricated using a modified sintering technique and two types of space holding agents (in vitro study)

**DOI:** 10.1038/s41405-025-00299-8

**Published:** 2025-02-20

**Authors:** Omnia Ghabour, Nahla Taha, Salma Aboul Gheit, Mona Mohy El Din

**Affiliations:** 1 Master’s Student, Department of Dental Biomaterials, Faculty of Dentistry, Alexandria University, Alexandria, Egypt; 2 Teaching Assistant of Dental Biomaterials, Department of Dental Biomaterials, College of Dentistry, Arab Academy for Science, Technology and Maritime Transport (AASTMT), El-Alamein, Egypt; 3 Modeling and Simulation Department, Advanced Technology and New Materials Research Institute, City of Scientific Research and Technological Applications, New Borg Al-Arab City, Alexandria, Egypt; 4 Department of Dental Biomaterials Department, Faculty of Dentistry, Alexandria University, Alexandria, Egypt

**Keywords:** Dental biomaterials, Maxillofacial surgery

## Abstract

**Objective:**

The aim of this work was to study the mechanical, degradation behavior and bioactivity of porous magnesium-based scaffolds alloyed with zinc and hydroxyapatite, fabricated using two different types of space holding agents and a modified powder metallurgy route.

**Methods:**

Powder particles of magnesium, zinc, hydroxyapatite (HA) and spacers were mixed, then mixtures were divided into 6 groups: IA (urea/0%HA), IB (urea/5%HA), IC (urea/7.5%HA), IIA (ammonium bicarbonate/0%HA), IIB (ammonium bicarbonate/5%HA) and IIC (ammonium bicarbonate/7.5%HA). A modified powder metallurgy route was used to fabricate the composites. Porosity analysis and microstructural characterization using Scanning Electron Microscope (SEM), Energy Dispersive X-ray Analysis (EDX), and X-ray Diffraction Analysis (XRD) were done. Evaluation of mechanical properties, in-vitro degradation rate in simulated body fluid (SBF) and in-vitro bioactivity using SEM and XRD were done. Data were statistically analyzed using two-way and three-way repeated ANOVA tests.

**Results:**

All scaffolds were found to be highly porous. Significant differences were observed regarding mechanical properties, degradation rate and concentration of magnesium released during degradation (*P*  < 0.0001). The results showed that group IIB had the lowest strength and fastest corrosion rate, while IB had the highest strength and elastic modulus and the slowest corrosion rate among all groups. Bioactivity evaluation revealed extensive formation of calcium phosphate crystals and precipitations covering the scaffolds’ surfaces.

**Conclusion:**

This study showed that using up to 5% HA as a reinforcing element with moderate compaction pressure and urea as a space holding agent can result in the fabrication of magnesium scaffolds suitable for orthopedic applications.

## Introduction

Bone defects are usually repaired using bone grafts such as autografts and allografts [1, 2]. However, due to their limited availability, researchers shifted towards using ceramics, polymers and metallic biomaterials from which bone scaffolds can be fabricated [1, 3, 4]. Bone scaffolds should be porous and biodegradable with adequate mechanical properties [5–7]. Ceramic scaffolds are brittle and polymers have low mechanical properties and low corrosion resistance which led researchers to turn their attention to using metals because of their superior mechanical properties and better corrosion resistance. Some of the commonly used metals are stainless steel, cobalt and titanium alloys [1, 5, 6, 8, 9]. However, these metals are not biodegradable and stress shielding of bone occurs because of the mismatch of their elastic moduli with that of bone [5, 6, 10]. These problems can be avoided by using biodegradable metallic biomaterials such as Magnesium (Mg) which provide short-term mechanical support for repair before their gradual degradation to be replaced by new bone formation [3, 5, 11].

Mg is considered a promising biomaterial for bone implants and scaffolds owing to its low density, elastic modulus which is close to the modulus of bone, its biocompatibility and spontaneous biodegradation in biological fluids [3, 5, 11–14]. However, the main drawback of pure Mg is its fast degradation in chloride-containing biological systems resulting in production of hydrogen gas which disrupts the initial healing process and causes the implant to deteriorate [10, 12]. Various methods have been proposed and investigated to slow down Mg corrosion and optimize its properties. [1, 4, 6]. One of these methods is alloying Mg with suitable alloying elements such as zinc. [5, 15]. Zinc (Zn) is considered a safe choice for biomedical applications and it has been established that using zinc can slow down Mg degradation and react with the generated hydrogen gas [1, 5]. Hydroxyapatite (HA) is another useful reinforcing element that can improve Mg bioactivity and slow down its degradation [1, 12].

Mg-based porous scaffolds can be fabricated using a number of techniques [1, 14, 16]. One of them is the powder metallurgy route that produces porous scaffolds using space holding agents such as carbamide (urea) and ammonium bicarbonate which are eliminated through thermal degradation leading to generation of pores [13, 15]. This technique involves mixing of powder particles then pressing them at a range of 100–500 MPa. Then, two-step sintering is done by heating to 130–250 °C with 2 to 5 h holding time at this temperature depending on the type of the used spacer. Then, heating to 500°–640 °C with a dwell time of 2 h [5, 6, 11, 17, 18].

This work aimed at using a modified powder metallurgy route to fabricate porous Mg-Zn-HA scaffolds that were alloyed with Zn and reinforced with different concentrations of hydroxyapatite. Porosity was created using two types of spacers (urea and ammonium bicarbonate). The modifications that were done in the powder metallurgy route included using a high pressure for pressing the powder particles of both the scaffolds that used urea and ammonium bicarbonate, and also using shorter sintering times and lower sintering temperatures in case of scaffolds that used urea. This was done as an attempt to replace the energy-intensive time-consuming fabrication procedures used in previous studies. After the fabrication, the effects of these modifications on the scaffolds’ properties were studied, comparing those fabricated using urea to those fabricated using ammonium bicarbonate. Characterization and evaluation of scaffolds included calculation of porosity percentage using the modified liquid displacement method, microstructural analysis using SEM, EDX and XRD. Then, compressive strength was measured and the corrosion behavior and bioactivity were evaluated by immersing the scaffolds in SBF for various durations. The null hypothesis of this study proposes that there is no significant difference between the properties of the scaffolds produced using urea and a modified powder metallurgy route and the properties of the scaffolds produced using ammonium bicarbonate.

## Materials and methods

This study received ethical approval and ethical clearance from the Faculty of Dentistry Research Ethics Committee at Alexandria University (0690/22-5-2023).

### Sample size calculation

In this in vitro study, the sample size was estimated assuming 80% study power and 5% alpha error (95% confidence interval) using G*Power Version 3.1.9.6 software. A total of 90 specimens were used. The study specimens were divided into 6 groups and the required sample size for each group was 15.

### Samples’ preparation

#### Synthesis of nanohydroxyapatite

HA was produced using wet-chemical precipitation method in which a solution of 0.5 M calcium nitrate tetrahydrate (Alfa Aesar, Germany) was added to a solution of 0.3 M ammonium phosphate dibasic (Alfa Aesar, Germany) at a 1:1 ratio until the formation of a white suspension. Gradual addition of ammonia was done until the pH of the solution reached 10 and was stirred for 3 h. The powder was sintered at 1000 °C in a muffle furnace (HD-150, Hobersal, Spain) [19].

#### Fabrication of the composites

Commercially available magnesium powder (SDFCL Sd Fine Chem Limited, India, purity ≥ 98%, average particle size 100 µm) and zinc powder (SDFCL Sd Fine Chem Limited, India, purity ≥ 99.5%, average particle size ≤ 45 µm) were used to fabricate magnesium-based composites. Carbamide (urea) powder (Alpha Chemika, India, purity ≥ 99.5%, average particle size 45–250 µm) and ammonium bicarbonate powder (PIOCHEM, Egypt, purity ≥ 99.5%, average particle size 45–250 µm) were used as space holding agents.

#### Powder Metallurgy Route

The powder particles were weighed and mixed using mortar and pestle according to the compositions in Table 1 until homogenous distribution of the alloying elements was obtained. Hydraulic press (GS25011 Specac Manual Hydraulic Press, UK) was used for uniaxial pressing of the powder mixtures at 680 MPa compaction pressure and 5 min dwell time to form green compacts of 13 mm diameter and 9 mm thickness. Then, two-stage sintering process was performed.

**Table 1:** Compositions of the fabricated scaffolds.

Group Name	Composition	Space holding agents	
		Carbamide (vol%)	Ammonium bicarbonate (vol%)
IA (Control)	Mg-4%Zn	40%	–
IB	Mg-4%Zn-5%HA	40%	–
IC	Mg-4%Zn-7.5%HA	40%	–
IIA (Control)	Mg-4%Zn	–	40%
IIB	Mg-4%Zn-5%HA	–	40%
IIC	Mg-4%Zn-7.5%HA	–	40%

For the specimens using urea as a spacer, two stages of sintering were done in a tubular furnace (Carbolite Tube Furnace, Germany) under controlled atmosphere of highly pure argon at 250 °C with a heating rate of 3 °C/min and holding time of 3.5 h to evaporate the carbamide particles, then the second stage at 380 °C with a 4 °C/min heating rate and 2 h dwell time to increase metal particles fusion. For the specimens that contained ammonium bicarbonate as a spacer, samples were heated at 130 °C for 2 h in a vacuum furnace (OVA031.XX3.5 Fistreem Vacuum Oven, UK) with 3 °C/min heating rate to remove the ammonium bicarbonate and then, samples were heated for 2 h in the tube furnace at 550 °C and a heating rate of 4 °C/min [18]. After sintering, the specimens were left to cool down to room temperature under the argon atmosphere.

### Characterization and evaluation of the fabricated composites

#### Porosity analysis

Modified liquid displacement method was used by immersing the scaffolds for 10 min in a 5 mL measuring bottle which contained ethanol until the air completely overflowed. Then, porosity was calculated using this equation: Porosity (%) = ((M2 - M3 - M_s_) / (M1-M3)) x 100% where M1 is the initial mass of the ethanol-filled bottle, M_S_ is the mass of the dry scaffold, M2 is the mass of the scaffold submerged in the bottle filled with ethanol, and M3 is the mass of the bottle after removal of the scaffold [20].

#### Microstructure characterization

Microstructure and surface morphology were observed using scanning electron microscope (SEM), (JSM-5, JEOL, Japan) at magnification x50. The pore diameters were measured using ImajeJ software (Version 1.54 g, https://imagej.nih.gov/ij). Energy Dispersive X-ray (EDX) (JSM-5, JEOL, Japan) was used for elemental analysis. The crystallographic phases were identified using X-ray Diffraction Analysis (XRD) (Shimadzu, XRD-7000, MAXima, Japan) [5, 13].

#### Evaluation of mechanical properties

Compressive strength and Young’s modulus were measured using universal testing machine (UTM) (5ST, Tinuis Olsen, England) on 8 mm × 12 mm cylinders with 0.5 mm/min crosshead speed according to ASTM E9-89a [21].

#### Immersion (in vitro degradation) testing and in vitro bioactivity evaluation

Static immersion testing was performed following ASTM-G31-72. Samples were weighed before immersion using a digital balance (AS 220-R2, RADWAG Wagi Electroniczne, Poland), then they were immersed for 7, 14, 28, 35 and 42 days in simulated body fluid (SBF) prepared according to Kokubo and Takadama [22]. Specimens were kept in plastic vials at 37 ± 1 °C in an incubator. To determine degradation rate, the incubated samples were retrieved after each time interval and corrosion products were cleaned with chromic acid prepared by mixing chromium trioxide **(**Oxford Lab Fine Chem LLP, India**)**, silver nitrate and barium nitrate (Alpha Chemika, India). Specimens were then washed with distilled water, and weighed to determine mass after immersion. The corrosion rate (C_R_) was calculated using the following equation: C_R_ = ∆m / (Axt) where C_R_ is the corrosion rate in mg cm^−2^ day^−1^, Δm is the weight loss in mg, *A* is the original surface area that was exposed to the corrosive solution in cm^2^ and *t* is the immersion time in days [10, 13].

Atomic absorption spectroscopy (AAS) (Shimadzu AA-7000, Japan) was used to determine the concentrations of Mg^2+^ ions which were released during immersion and corrosion morphologies of retrieved samples were observed using SEM [10].

In-vitro bioactivity was evaluated after 14 and 28 days of immersion. Crystallographic phases of corrosion products and calcium phosphate crystals formed after immersion were detected and recorded using XRD [23]. Surface morphology was viewed using SEM [24]. Types of formed crystals, their quantity and degree of crystallinity were determined using QualX software (Version 2.24, https://www.ba.ic.cnr.it/softwareic/qualxweb/).

### Statistical analysis

The normal distribution of the data was confirmed through the application of the Shapiro–Wilk test and examination of Q–Q plots. Descriptive statistics, including mean, standard deviation (SD), and 95% confidence interval (CI), were utilized for data presentation. Two-way ANOVA was employed to evaluate the impact of space-holding agents and Mg-Zn-HA scaffolds containing different concentrations of hydroxyapatite (HA) on mechanical parameters such as stress, elastic modulus, and porosity of the scaffolds. Additionally, a three-way repeated measures ANOVA was conducted to discern the combined effects of immersion time, space-holding agents and Mg-Zn-HA scaffolds containing different HA concentrations on degradation rate and Mg concentrations. Post-hoc pairwise comparisons were carried out, and *p* values adjustments were made using the Bonferroni method. All tests were two tailed and the significance level was set at *p* value ≤ 0.05. Data were analyzed using IBM SPSS, version 23 for windows, Armonk, NY, USA and graphical presentation was done using GraphPad Prism version 10.0.0 for Windows, GraphPad Software, Boston, Massachusetts USA, www.graphpad.com.

## Results

### Porosity analysis

**Fig. 1:**
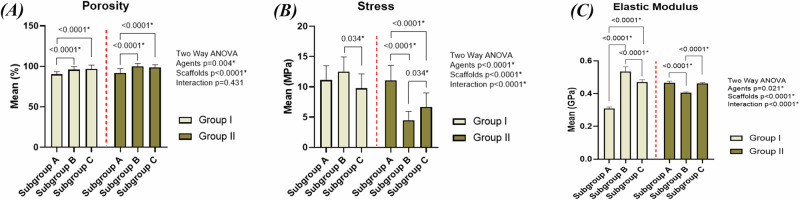
Graphical illustration of the data obtained from porosity analysis and mechanical testing. (**A**) porosity (%), (**B**) compressive strength (MPa) and (**C**) elastic modulus (GPa) of the scaffolds.

The percentage porosities for groups IA, IB, IC, IIA, IIB and IIC were found to be 90.3%, 95.9%, 96.85%, 91.7%, 99.9% and 98.9%, respectively. The percentage porosity of specimens fabricated using ammonium bicarbonate was higher than those using urea. Adding HA in groups IB, IC, IIB and IIC was accompanied by an increase in percentage porosity with groups IIB and IIC having the highest porosity among all scaffolds. The two-way ANOVA (Fig. 1A and supplementary table 2) indicates that there is no significant interaction between the different types of space holding agents and different concentrations of HA (*F* = 0.85, *P* = 0.431, η2  = 0.020). However, each of the two variables has significant effect on the percentage porosity individually (for the different types of space holding agents: *F* = 8.58, *P*  = 0.004, η2  = 0.093 and for the different concentrations of hydroxyapatite: *F* = 29.35, *P*  <  0.0001, η2  = 0.411). Pairwise comparisons between study groups regarding porosity are presented in supplementary table 3.

### Microstructure characterization of the sintered specimens

**Fig. 2:**
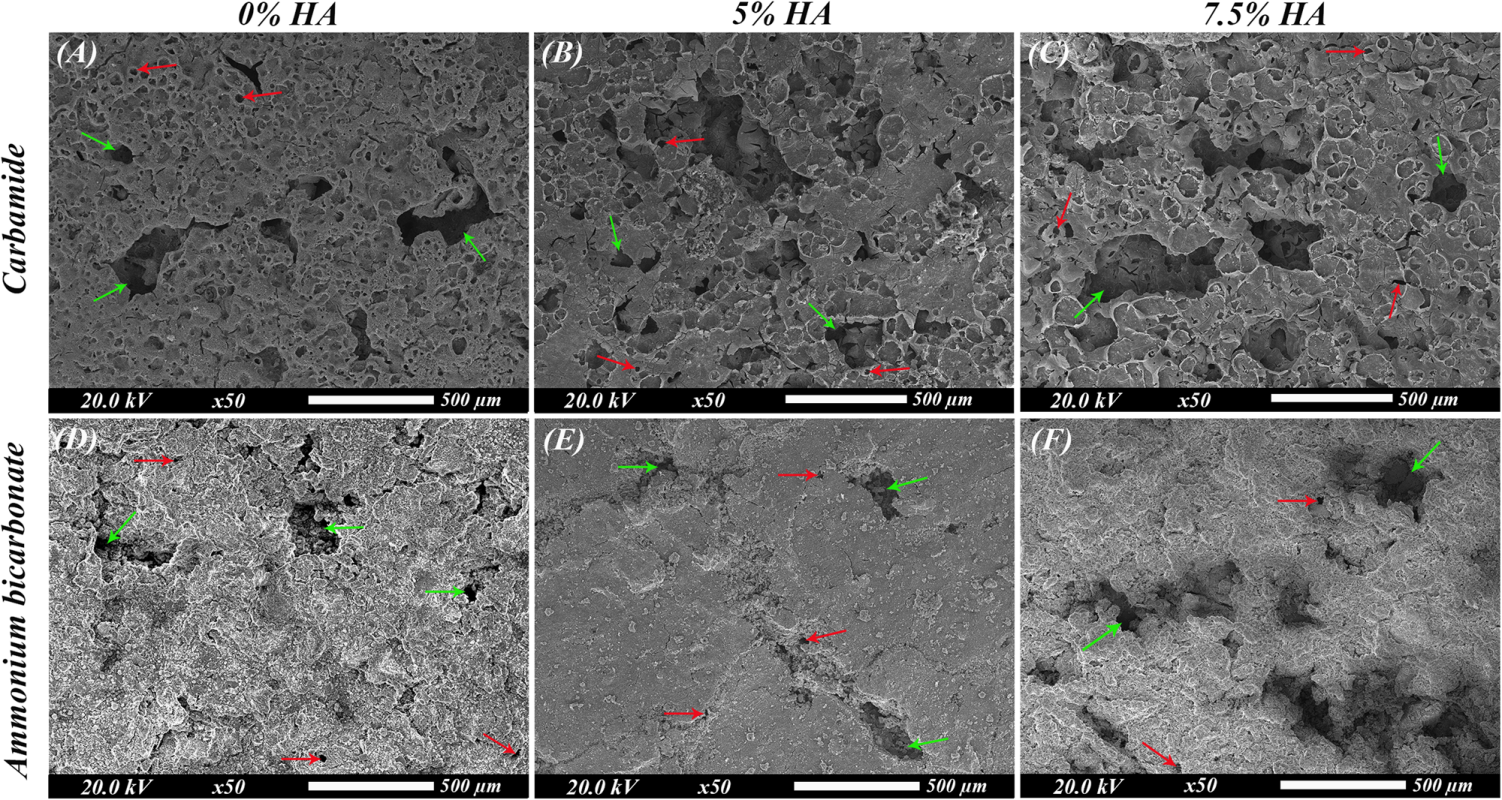
SEM images of the sintered scaffolds. **A** Group IA (Mg-4%Zn with urea as a spacer), **B** Group IB (Mg-4%Zn-5%HA with urea as a spacer), **C** Group IC (Mg-4%Zn-7.5%HA with urea as a spacer), **D** Group IIA (Mg-4%Zn with ammonium bicarbonate as a spacer), **E** Group IIB (Mg-4%Zn-5%HA with ammonium bicarbonate as a spacer) and **F** Group IIC (Mg-4%Zn-7.5%HA with ammonium bicarbonate as a spacer). Red arrows denote micropores and green arrows denote macropores.

**Fig. 3:**
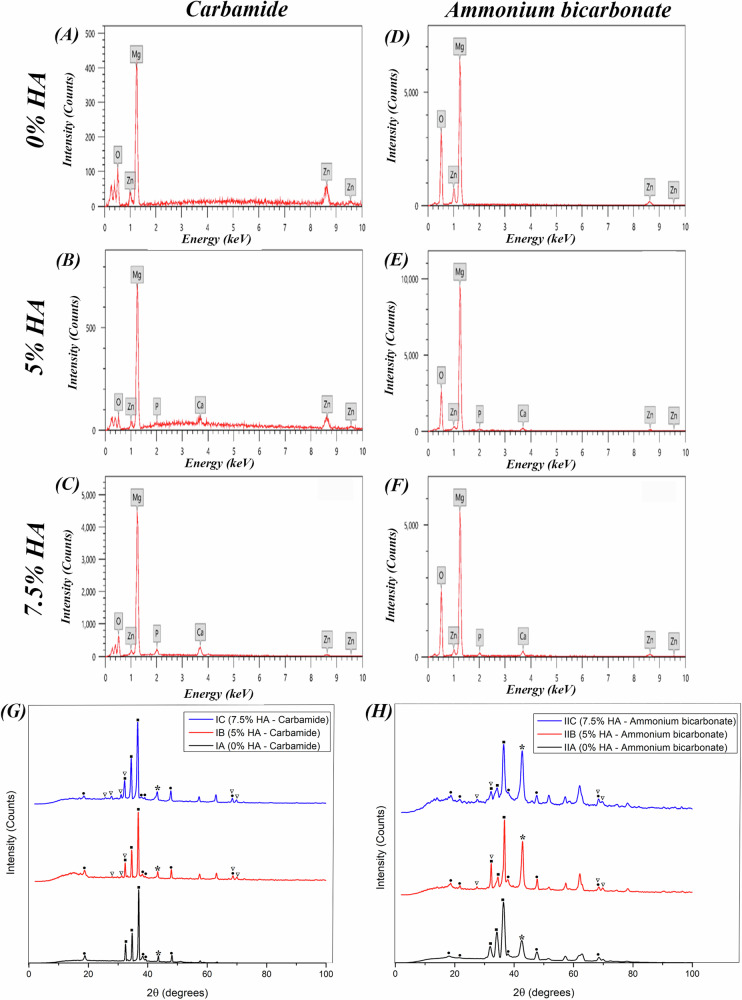
EDX spectra and XRD patterns of the sintered scaffolds. **A**–**F** EDX analysis of the sintered scaffolds: **A** Group IA (Mg-4%Zn with urea as a spacer), **B** Group IB (Mg-4%Zn-5%HA with urea as a spacer), **C** Group IC (Mg-4%Zn-7.5%HA with urea as a spacer), **D** Group IIA (Mg-4%Zn with ammonium bicarbonate as a spacer), **E** Group IIB (Mg-4%Zn-5%HA with ammonium bicarbonate as a spacer) and **F** Group IIC (Mg-4%Zn-7.5%HA with ammonium bicarbonate as a spacer). **G** XRD patterns of groups IA, IB and IC. **H** XRD patterns of groups IIA, IIB and IIC. The ■ indicates Mg peaks, the * indicates the MgO peaks, the • indicates Mg-Zn peaks and the ▽ indicates HA peaks.

The microstructure of scaffolds is shown in SEM images (Fig. 2A–F). The presence of irregular spherical and elliptical flattened pores distributed all over the surface confirms the highly porous structure of the scaffolds. Two types of pores could be observed in all the scaffolds. The interconnected macropores with pore size ranging from 50 to 500 µm and the isolated micropores which are less than or equal to 50 µm. Mg, Zn, Calcium (Ca), Phosphate (P) and Oxygen (O) elemental peaks could be observed in the EDX spectra (Fig. 3A–F). As the percentage of HA increases from 5% in groups IB and IIB to 7.5% in groups IC and IIC, the intensity of Ca and P peaks increases. XRD phase analysis can be observed in Fig. 3G, H. The XRD patterns reveal the presence of peaks corresponding to Mg, Mg-Zn and MgO phases in all specimens. Characteristic peaks of HA can be clearly identified. The intensity of MgO peaks was higher in scaffolds using ammonium bicarbonate as a spacer compared to those using carbamide.

### Mechanical characterization

**Fig. 4:**
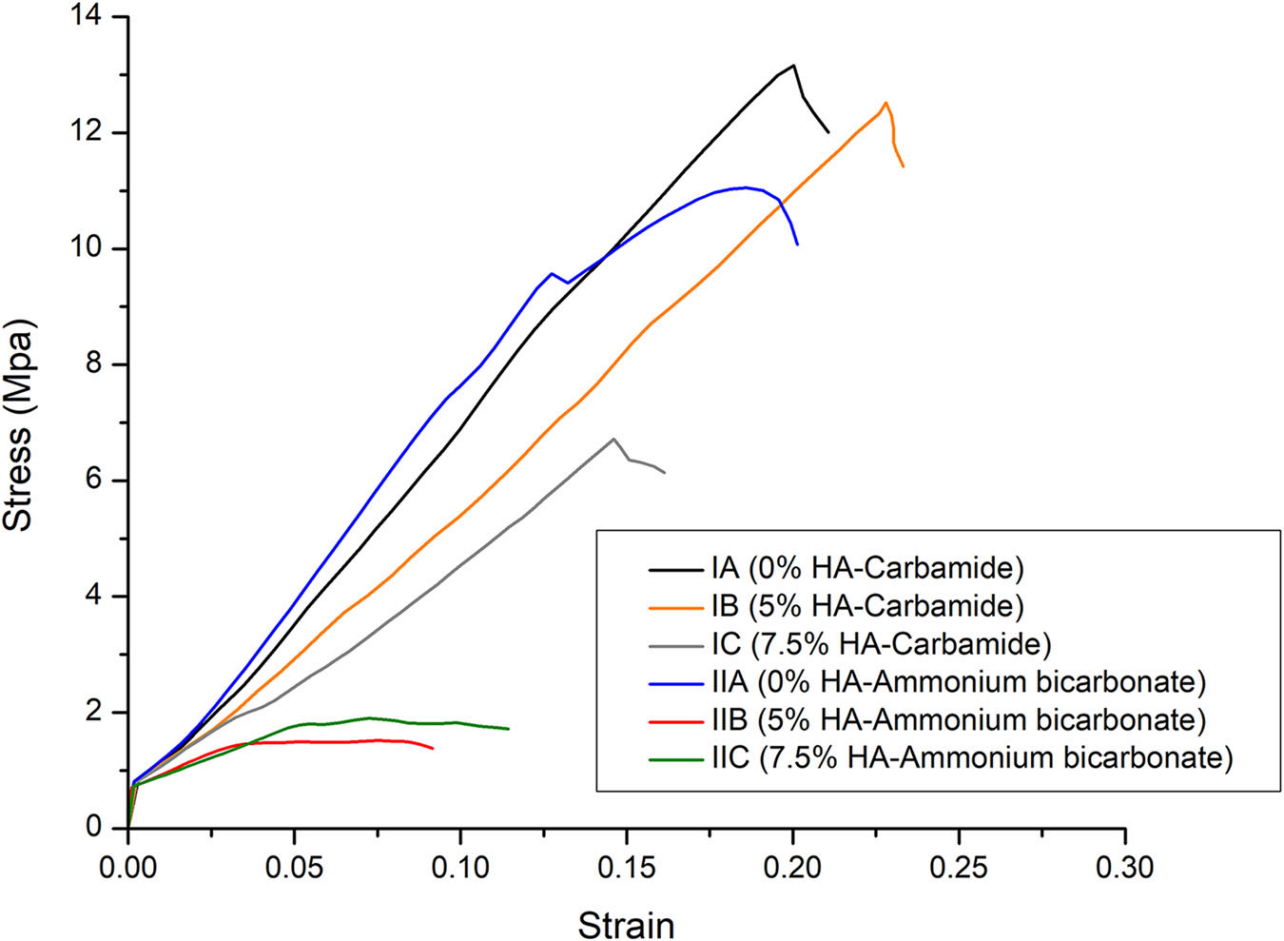
Stress-strain curves of the sintered specimens obtained from the compressive strength testing.

Upon observing stress-strain curves (Fig. 4), the curves of IA, IB and IC consisted of the following zones: a small linear elastic region, followed by a zone in which the stress increased till the ultimate strength was reached, then a third zone showing a sudden drop in the stress which indicates brittle fracture of the specimens. Upon comparing the values of ultimate compressive strength and elastic moduli of groups IA, IB and IC (Fig. 1B, C and Supplementary Table 1), specimens of IB had higher values than those of group IA. However, marked decrease in strength and elastic modulus in group IC was observed.

In groups IIA, IIB and IIC, the following zones were found in the curves of IIA: a small linear elastic region, then increase in stress, then slight drop in stress, followed by another zone of stress increase, then stress decreased again till the fracture point was reached. However, groups IIB and IIC had these zones: a linear elastic region, a zone of slight increase in the stress and a plateau region which indicates collapsing and fracture of the walls under an almost constant load with increasing strain till the point of complete failure was reached. When comparing the ultimate compressive strength and elastic moduli of groups IIA, IIB and IIC to each other (Fig. 1B, C and Supplementary Table 1), it was found that group IIA had the highest values of strength and elastic modulus followed by IIC then IIB.

The two-way ANOVA indicates that there is significant interaction between the different types of space holding agents and different concentrations of hydroxyapatite leading to significant effect on strength (Fig. 1B and supplementary table 4) (*F* = 23.10, *P*  < 0.0001, η2  = 0.355) and elastic modulus (Fig. 1C and supplementary table 6) (*F* = 708.93, *P*  < 0.0001, η2  = 0.944) of the scaffolds. Pairwise comparisons between study groups regarding Stress and elastic modulus are presented in supplementary tables 5 and 7, respectively.

### Immersion (in vitro degradation) testing

**Fig. 5:**
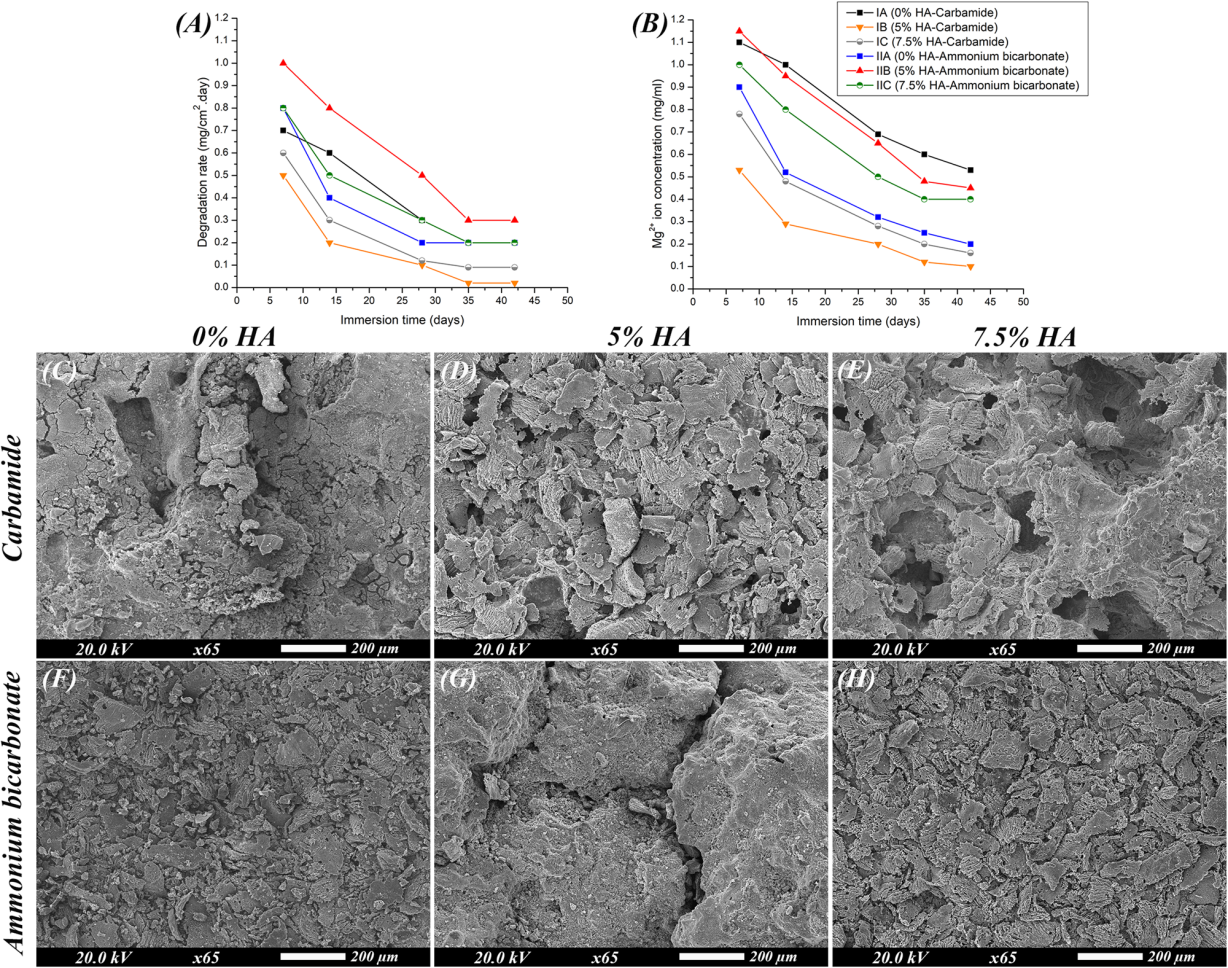
The calculated degradation rates, the measured magnesium ion concentrations released during degradation and representative SEM images of the scaffolds’ corrosion morphologies. **A** Degradation rates of the scaffolds after immersion for different periods of time (7, 14, 28, 35 and 42 days) calculated by the weight loss method. **B** Concentration of Mg^2+^ ions released during immersion testing which were determined through atomic absorption spectroscopy (AAS) after immersion for different periods of time (7, 14, 28, 35 and 42 days). **C**–**H** SEM images representing the corrosion morphologies of the scaffolds after 42 days of immersion: **C** Group IA (Mg-4%Zn with urea as a spacer), **D** Group IB (Mg-4%Zn-5%HA with urea as a spacer), **E** Group IC (Mg-4%Zn-7.5%HA with urea as a spacer), **F** Group IIA (Mg-4%Zn with ammonium bicarbonate as a spacer), **G** Group IIB (Mg-4%Zn-5%HA with ammonium bicarbonate as a spacer) and (**H**) Group IIC (Mg-4%Zn-7.5%HA with ammonium bicarbonate as a spacer).

All groups show a similar tendency for corrosion by having high corrosion rate during the first periods of immersion which then decreased till it became constant after 35 days of immersion (Fig. 5A). Groups IB and IC had slower corrosion rates than IA. However, group IC had higher corrosion rates than IB despite being reinforced with more HA. Regarding groups IIA, IIB and IIC, group IIB had the highest corrosion rates and the rate of corrosion of IIC was found to be close to or slightly higher than that of IIA. The concentrations of Mg^2+^ ions released in the SBF are shown in Fig. 5B. For all groups, Mg^2+^ concentration was initially high, then it decreased over time with IA, IIA, IIB and IIC releasing higher amounts of Mg^2+^ ions compared to other groups. The three-way repeated measures ANOVA indicates that using different types of space holding agents, using different concentrations of hydroxyapatite and the increase in immersion time have significant effect on the degradation rate of the scaffolds (Table 2) (*F* = 18.19, *P*  < 0.0001, η2  = 0.302) and concentration of the released Mg^2+^ ions (Table 3) (*F* = 15.95, *P*  < 0.0001, η2  = 0.275). Pairwise comparisons between study groups regarding degradation rate are presented in supplementary tables 8a and 8b and regarding Mg^2+^ concentration in Supplementary Table 9a and 9b.

**Table 2:** Three-way repeated measures ANOVA assessing the effect of different parameters on degradation rate of scaffolds.

Parameters	Mean Square	F test	*p* value	Partial Eta Squared
**Time**	6.74	892.58	<0.0001*	0.914
**Space holding agents**	3.22	500.32	<0.0001*	0.856
**Mg-Zn-HA scaffolds with different HA concentrations**	0.22	33.85	<0.0001*	0.446
**Space holding agents × Mg-Zn-HA scaffolds with different HA concentrations**	2.14	332.77	<0.0001*	0.888
**Time x space holding agents**	0.07	9.45	<0.0001*	0.101
**Time x Mg-Zn-HA scaffolds with different HA concentrations**	0.02	2.99	0.006*	0.066
**Time x space holding agents × Mg-Zn-HA scaffolds with different HA concentrations (Interaction)**	0.14	18.19	<0.0001*	0.302

^*^Statistically significant difference at *p* value ≤ 0.05.

**Table 3:** Three-way repeated measures ANOVA assessing the effect of different parameters on the released Mg^2+^ concentration.

Parameters	Mean Square	F test	*p* value	Partial Eta Squared
**Time**	7.29	1101.63	<0.0001*	0.929
**Space holding agents**	1.80	223.89	<0.0001*	0.727
**Mg-Zn-HA scaffolds with different HA concentrations**	0.67	83.03	<0.0001*	0.664
**Space holding agents × Mg-Zn-HA scaffolds with different HA concentrations**	6.94	864.51	<0.0001*	0.954
**Time x space holding agents**	0.11	15.80	<0.0001*	0.158
**Time x Mg-Zn-HA scaffolds with different HA concentrations**	0.01	2.19	0.041*	0.050
**Time x space holding agents × Mg-Zn-HA scaffolds with different HA concentrations (Interaction)**	0.11	15.95	<0.0001*	0.275

^*^Statistically significant difference at *p* value ≤ 0.05.

The scaffolds corrosion morphologies are represented in SEM images (Fig. 5C–H). Corrosion of scaffolds appears in the form of flaking, disintegration of scaffold matrices and micro-cracking of the surface together with formation of holes and fissures. Specimens of groups IB and IC appear to be less corroded than those of IA which have more fissuring. However, group IC shows the formation of numerous large pits compared to IB. The degradation morphologies of groups IIA, IIB and IIC show that IIB and IIC appear to be more corroded than IIA with large deep cracking present on the surfaces of IIB and more flaking and numerous holes present in IIC.

### In-vitro bioactivity

**Fig. 6:**
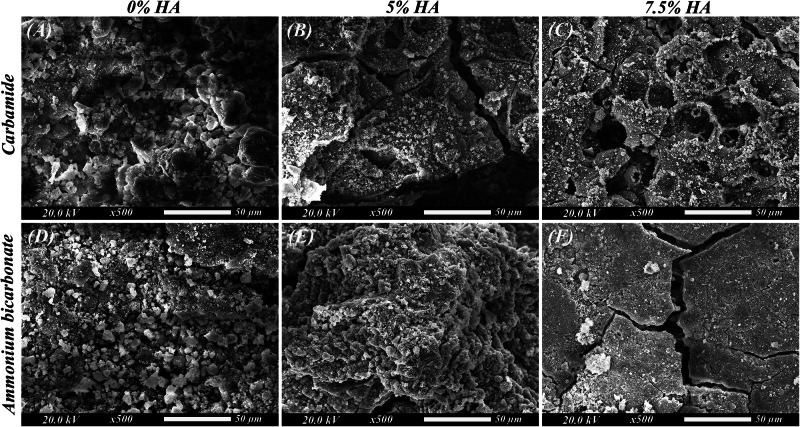
SEM images of the scaffolds after 14 days of immersion. **A** Group IA (Mg-4%Zn with urea as a spacer), **B** Group IB (Mg-4%Zn-5%HA with urea as a spacer), **C** Group IC (Mg-4%Zn-7.5%HA with urea as a spacer), **D** Group IIA (Mg-4%Zn with ammonium bicarbonate as a spacer), **E** Group IIB (Mg-4%Zn-5%HA with ammonium bicarbonate as a spacer) and **F** Group IIC (Mg-4%Zn-7.5%HA with ammonium bicarbonate as a spacer).

**Fig. 7:**
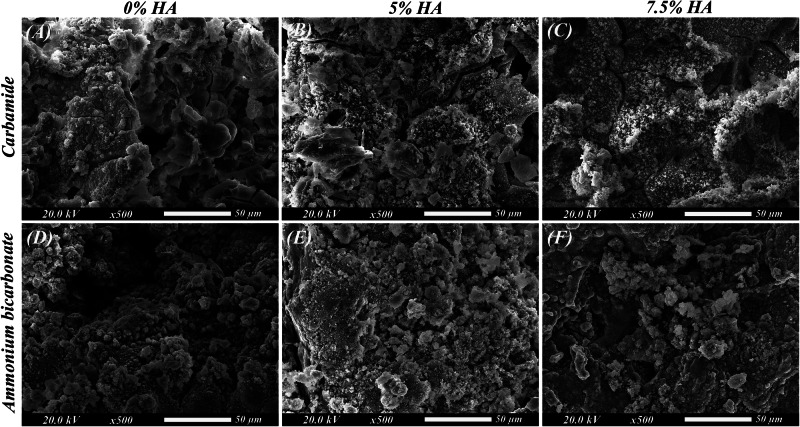
SEM images of the scaffolds after 28 days of immersion. **A** Group IA (Mg-4%Zn with urea as a spacer), **B** Group IB (Mg-4%Zn-5%HA with urea as a spacer), **C** Group IC (Mg-4%Zn-7.5%HA with urea as a spacer), **D** Group IIA (Mg-4%Zn with ammonium bicarbonate as a spacer), **E** Group IIB (Mg-4%Zn-5%HA with ammonium bicarbonate as a spacer) and **F** Group IIC (Mg-4%Zn-7.5%HA with ammonium bicarbonate as a spacer).

**Fig. 8:**
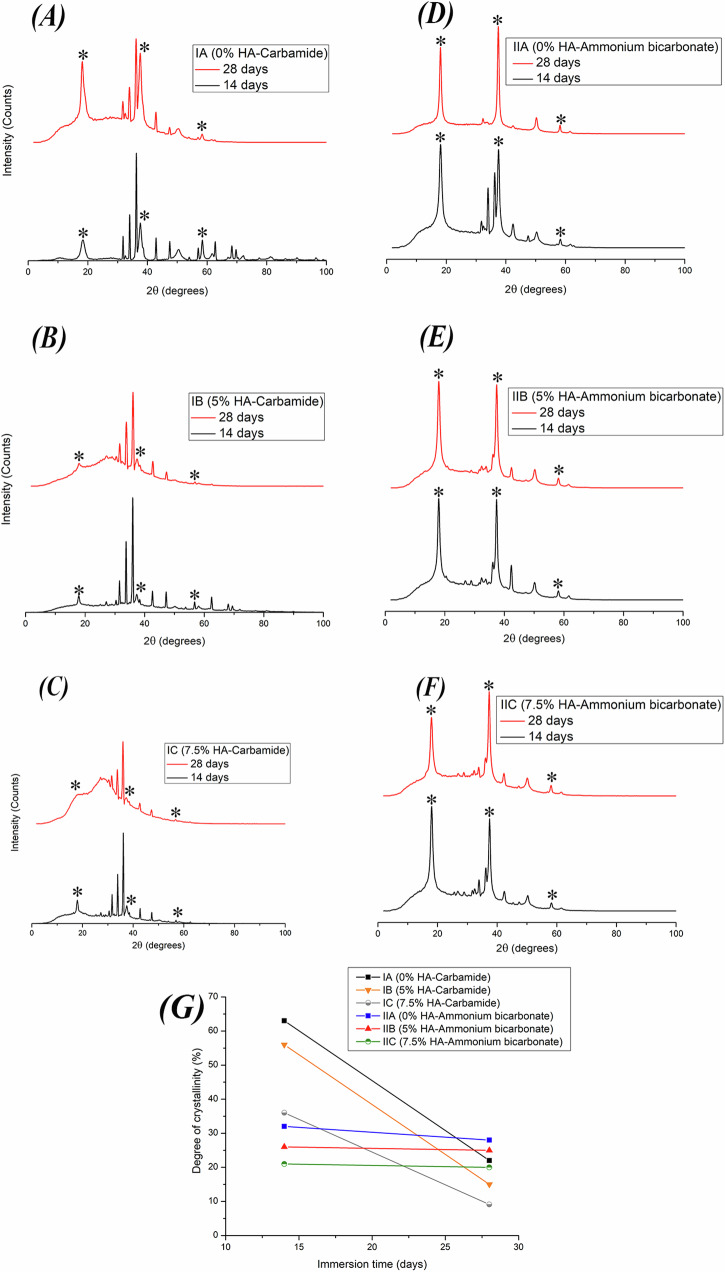
XRD patterns of the scaffolds after immersion in the SBF solution for 14 days and 28 days and a representative graph of the percentage of the degree of crystallinity after 14 and 28 days of immersion. **A**–**F** XRD patterns of the scaffolds after immersion in the SBF solution for 14 days and 28 days: **A** Group IA (Mg-4%Zn with urea as a spacer), **B** Group IB (Mg-4%Zn-5%HA with urea as a spacer), **C** Group IC (Mg-4%Zn-7.5%HA with urea as a spacer), **D** Group IIA (Mg-4%Zn with ammonium bicarbonate as a spacer), **E** Group IIB (Mg-4%Zn-5%HA with ammonium bicarbonate as a spacer) and **F** Group IIC (Mg-4%Zn-7.5%HA with ammonium bicarbonate as a spacer). The * symbol indicates Mg(OH)_2_ peaks. **G** Percentage of the degree of crystallinity after 14 and 28 days of immersion.

After 2 and 4 weeks of immersion, it can be observed in the SEM images (Figs. 6 and 7) that the surfaces of all specimens show irregular spherical calcium phosphate crystal precipitation. XRD analysis (Fig. 8A-F) conducted after 2 and 4 weeks of immersion showed different peaks corresponding to magnesium hydroxide (Mg(OH)_2_) formation which is the main corrosion product.

Table 4 shows some of the formed calcium phosphate phases with their quantities over 2 and 4 weeks of immersion. These phases were identified using the QualX software. It should be pointed out that for groups IA, IB and IC, few calcium phosphate phases were formed by the end of the 14-day immersion period but more phases were found after 28 days of immersion. Also, HA increases over time and for groups IA, IB and IC, which did not have HA after 2 weeks, HA was found after 4 weeks of immersion. It was found that for all groups, the degree of crystallinity decreased as the immersion time increased from 2 to 4 weeks (Fig. 8G). However, the decline in the degree of crystallinity was minimal for groups IIA, IIB and IIC compared to the marked decrease in IA, IB and IC.

**Table 4:** Examples for the formed calcium phosphate phases and their quantities identified using QualX software.

Phase (%)	After 2 weeks of immersion						After 4 weeks of immersion					
	IA	IB	IC	IIA	IIB	IIC	IA	IB	IC	IIA	IIB	IIC
Hydroxyapatite (Ca_10_(PO_4_)_6_(OH)_2_)	–	–	–	13.1	17.8	18.1	13.5	4.5	19.5	24.3	18.9	18.5
Tricalcium phosphate (Ca_3_(PO_4_)_2_)	–	–	–	13.7	13.6	13.5	18.6	24.1	14.7	12.4	14.7	13.5
Octa-calcium phosphate (Ca_8_H_2_(PO_4_)_6_.5H_2_O)	–	–	–	1.8	1.9	1.8	2.3	–	–	–	1.7	1.8
Calcium polyphosphate	–	–	–	1.7	2	2	2.6	4.2	3.2	1.3	1.8	1.9
Ca(PO_3_)_2_	–	–	30	12.1	12	10.6	13.5	15.2	15.1	10.7	10.8	10.5
Potassium calcium phosphate (KCaPO_4_.H_2_O)	–	–		7.8	8.4	8	–	7.6	4.7	12.7	9.3	7.1
Sodium calcium magnesium phosphate Na_8_Ca_1.5_Mg_12.5_(PO_4_)_12_	–	–	–	25.1	26.5	29.4	30.9	27	25.1	32.5	25.8	28.2
Chlorapatite Ca_10_(P O_4_)_6_Cl_2_	100	–	–	2	3.6	3.5	4.2	3.3	3.4	3.6	3.7	5
KNa_4_Ca(Mn_10_Fe_4_)Al(OH)_2_(PO_4_)_12_	–	–	70	11.6	14	13.1	14.2	13.9	14.3	13.8	13.2	13.4

## Discussion

In this work, powder metallurgy route was used to fabricate magnesium-based scaffolds that were reinforced with zinc and hydroxyapatite and using two types of space holding agents. Our aim was to compare between the properties of the scaffolds that used urea and the ones that used ammonium bicarbonate as space holding agents. Also, a modified powder metallurgy route was tried in which higher compaction pressure was used for all groups and lower sintering temperature and shorter sintering time were used in specimens that used urea. Also, we evaluated the effect of adding hydroxyapatite on the properties of the scaffolds. Manual mixing using mortar and pestle was chosen as the mixing method in this study for being an accessible and affordable method of mixing in comparison to mechanical methods of mixing. In order to use a mechanical mixing method, it is necessary to have optimal mixing and milling parameters to have homogenous mixtures and good scaffold properties [25] and this was not available for our study and was costly and time consuming. Therefore, mechanical mixing was not used because if the mixing parameters were not ideal, mechanical mixing can lead to many problems such as agglomerations of the particles resulting in inhomogeneous mixtures [25]. Also, it leads to temperature generation which can lead to formation of new phases changing the composition and properties of the scaffolds [26, 27]. The null hypothesis was rejected as there was significant difference between the groups using different space holding agents and different concentrations of hydroxyapatite.

Percentage of porosity and pore spaces of the scaffolds were determined using modified liquid displacement method. The difference in melting temperatures of Mg (has low melting temperature) and HA (has high melting temperature) [28] led to difficulty of fusion of these particles at low sintering temperatures (380 °C and 550 °C) used during scaffold fabrication. Consequently, improper sintering, decreased densification, and increased porosity were found in scaffolds containing HA [28, 29]. The high porosity of scaffolds with ammonium bicarbonate spacers is attributed to the high compaction pressure (680 MPa) [30]. A high compaction pressure is beneficial as it breaks down the oxide layer on the surfaces of metallic particles leading to improved bonding and densification [8]. However, such pressure exceeded the compressive strength of the spacer particles leading to severe plastic deformation and changes in their morphology creating interconnected pores and in turn increases the porosity [30, 31].

Pore morphology shown in SEM images (Fig. 2A–F) resembles the rod-shaped irregular strips of urea and the irregular spherical cubic ammonium bicarbonate. Macropores are formed after the removal of the spacers by thermal decomposition during the first step of sintering or due to gaseous expansion after evaporation of the spacer [13]. These pores range from 50-500 µm which is the optimum diameter required for proper attachment and diffusion of bone forming cells into the scaffold matrix [32, 33]. Difference in rates of diffusion of Mg and Zn atoms when heated during sintering leads to volume reduction (Kirkendall effect) which forms micropores [13].

According to the XRD findings, the presence of Mg-Zn intermetallic phases at temperatures as low as 380 °C denotes that there is a reaction between Mg and Zn powder particles at this temperature. Adding 4% Zn contributes to grain refinement and formation of Mg-Zn phases which improve the strength by dispersion strengthening mechanism [6]. The increased intensity of MgO peak in IIA, IIB and IIC implies that these groups have undergone more oxidation than IA, IB and IC during the sintering process which is believed to be due to the higher sintering temperature (550 °C) used in IIA, IIB and IIC [6]. However, MgO has been found to be a biologically stable phase that will not cause any harmful reactions in the body [34].

Regarding the mechanical properties of groups IA, IB and IC, the high strength and elastic modulus of IB compared to IA can be attributed to the grain refinement resulting from the 5% HA added to reinforce the scaffolds of group IB [5]. Additionally, the high elastic modulus of HA imparts an increase of stiffness in reinforced scaffolds. Surprisingly, a decline in strength and elastic modulus is found in group IC despite having more HA. By increasing the HA percentage, agglomeration of the particles occurs acting as stress concentration areas increasing scaffold brittleness [10, 21]. Based on the aforementioned findings and agreeing with the results of some previous research [10, 21], it is recommended to add HA up to 5 wt.%.

For groups IIA, IIB and IIC, group IIA had higher strength and elastic modulus values compared to HA reinforced groups. In this work, upon observing the MgO peak in XRD spectra, it was found that its intensity significantly increased in IIB and IIC compared to its intensity in IIA. MgO, which is a highly stable phase with high melting point, hinders the diffusion and bonding of Mg particles during sintering leading to the formation of micropores which increases the porosity; therefore, leading to a decline in scaffold strength of groups IIB and IIC [15, 35]. Also, irregular, sharp, elliptical pores such as those observed in the highly porous scaffolds of IIB and IIC act as stress concentration areas leading to initiation, propagation of cracks and brittle fracture of the porous structure [15, 18, 36–38]. Group IIC has higher mechanical properties than IIB as the foams of IIB have undergone more oxidation and have more porosity than those of IIC. It can be stated that for foams of IIB and IIC, the effect of oxidation and porosity was more prominent compared to the influence of alloying elements. This also explains the higher strength of IB and IC compared to IIB and IIC which are more porous and oxidized. It is worth noting that the mechanical properties of these Mg composites are within the range of those of cancellous bone as the compressive strength of cancellous bone is 0.2–80 MPa and its elastic modulus is 0.01–2 GPa [5] and despite the strength reduction, the high porosity can enhance mechanical interlocking between the implanted foam and the adjacent tissue, increasing implant stability [39].

Corrosion behavior of the composites was evaluated by in-vitro degradation test and the weight loss method was used to determine the rate of degradation. The data in Fig. 5A show fast degradation in initial stages because large surface areas of the scaffolds are exposed to solution and not yet covered by any precipitates. Interaction between solution and scaffold leads to formation of Mg(OH)_2_ and apatite crystals that act as a barrier against the corrosive effect of the SBF which slows down the degradation rate till a constant rate is reached [21]. Degradation rates are affected by microstructure and pore characteristics because pores act as a passage through which corrosive ions from the solution (chlorine Cl^**-**^) travel to reach deep areas of the scaffold matrix and accumulate inside the pores [21]. The ion attack leads to disintegration of scaffold and apatite layers which expose new layers to be attacked by more ions leading to an accelerated degradation rate [15, 21, 40]. Therefore, groups with higher porosity had faster degradation rates.

The slower corrosion rates of IB and IC compared to group IA can be attributed to the influence of HA which is a bioactive mineral that provides the scaffold with nucleation sites for precipitation of calcium phosphate crystals, facilitating growth of thick apatite layers on scaffold surfaces [21, 24]. These apatite layers are more stable than Mg(OH)_2_ which undergo dissolution due to formation of soluble magnesium chloride (MgCl_2_) [40, 41]. Group IC degrades faster than group IB due to its increased porosity and HA concentration [17, 21]. For groups IIB and IIC, HA addition was not effective in decreasing degradation rate compared to group IIA because of the higher porosity and lower strength of IIB and IIC. Moreover, because of this higher porosity and lower strength of IIB and IIC, their degradation rate was faster than IB and IC as groups IB and IC in which urea spacers were used had lower porosity and higher strength, and, as a result, slower degradation rates than groups IIB and IIC in which ammonium bicarbonate spacers were used.

The rate and concentration of released Mg^2+^ ions decrease over time because of the decreased corrosion rate caused by the formation of protective apatite and corrosion products. Mg^2+^ ion concentration is higher in specimens that have high degradation rates such as groups IA, IIA, IIB and IIC.

SEM images of corrosion morphologies (Fig. 5C-H) show that all scaffolds have undergone corrosion manifested in the form of flaking and cracking of their surfaces. It should be highlighted that HA reinforcement increased the corrosion resistance which is manifested by the lower number of cracks present in IB and IC compared to IA. The large deep cracking found in group IIB is consistent with IIB having the highest degradation rate. Specimens of groups IIB and IIC appear to be more corroded than those of IIA which is also consistent with the findings of their degradation rates.

Regarding the in-vitro bioactivity, lots of precipitations and particle deposition can be observed (Figs. 6 and 7) proving the scaffolds to be bioactive as magnesium is a potent bioactive and biocompatible element, so the released Mg^2+^ ions interact with the physiologic environment enhancing cell attachment and proliferation and facilitating formation of calcium phosphates needed for bone mineralization [42, 43]. Moreover, HA can also induce deposition of bone-like apatite due to its bioactivity and osteoconductivity [24].

The chemical formula of apatite is M_10_(PO_4_)X_2_, a variety of different ions can occupy the X and M positions of the formula. For example, Ca^2+^, Mg^+^, Na^+^, Mn^2+^ can occupy the M position and Cl^-^ and H_2_O can occupy the X position [44]. These phases can harmonize with the apatite crystals of natural bone as the natural apatite can tolerate ionic substitutions and a lot of ions that are circulating in the bloodstream can be adsorbed on the surface and be a part of the bone mineral during its formation [44, 45]. Octa-calcium phosphate has been found to be a transient phase that can act as precursor for HA formation as these phases can aggregate together, attract new calcium and phosphate ions and get converted into HA crystals [46] and this is shown in Table 4 as it can be observed that as immersion time proceeds from 2 to 4 weeks, octa-calcium phosphate phase disappears or decreases and the amount of HA increases. Other phases such as the tricalcium phosphate and monohydrate calcium phosphate have been found to be osteoconductive and can promote bone growth [47].

After 2 weeks of immersion, few calcium phosphate phases have been formed in groups IA, IB and IC compared to IIA, IIB and IIC. This is probably because urea used in groups IA, IB and IC is a charge-neutral molecule [48] so any remnants of urea on scaffold surfaces can delay calcium-phosphate ion precipitation. On the other hand, ammonium bicarbonate is an ionic compound with +1 charge on the cation (Ammonium, NH_4_) and -1 charge on the anion (Bicarbonate, HCO_3_) [49], so even if there are remnants of ammonium bicarbonate, it will undergo quick ionization leading to faster attraction and precipitation of calcium-phosphate ions on scaffold surfaces of groups IIA, IIB and IIC.

The decrease in degree of crystallinity can be attributed to the degradation process which led to loss of ions and crystalline phases [50] as well as the formation of new amorphous calcium phosphate phases. The loss of crystalline phases and the formation of amorphous phases is more evident in groups IA, IB and IC and this explains the severe decrease in their degree of crystallinity compared to the minimal decrease in groups IIA, IIB and IIC.

This study has been found to have some limitations such as being an in-vitro study, so some differences may be found regarding the degradation behavior when these scaffolds are applied in vivo. Also, the mixing method may not be accurate and the processing parameters of the powder metallurgy route such as the compaction pressure need to be optimized.

## Conclusion

In this study, successful fabrication of magnesium-based scaffolds using urea and ammonium bicarbonate spacers and a modified powder metallurgy method was achieved. Adding HA improved mechanical properties and corrosion resistance, but its concentration and distribution are critical factors that can negatively affect scaffold properties as it was found that HA concentrations more than 5% had negative effects on the properties of the scaffolds. Also, processing parameters influence scaffold properties as high compaction pressure used in this work increased the porosity, decreased the strength, and increased the degradation rate of scaffolds that used ammonium bicarbonate. However, the scaffolds that used urea had higher mechanical properties and slower degradation rates because of having less porosity. Regardless, all composites had pore sizes suitable for attachment of osteoblasts and their mechanical properties were close to those of cancellous bone. Additionally, they showed remarkable bioactivity with the ability to induce apatite crystal precipitation. Also, it is worth noting that the lower sintering temperature and shorter sintering time used in case of urea did not have negative effect on the scaffolds proving that a more economical shorter powder metallurgy route can be used. Therefore, these scaffolds have the potential to be used in critical-sized bony defects as they can promote hard tissue regeneration.

## Supplementary information

Supplementary material

## Data Availability

Data are available upon request from the corresponding author.
