# Supplementary material for: Evaluation of magnesium-based scaffolds fabricated using a modified sintering technique and two types of space holding agents (in vitro study)
Source: BDJ Open. 2025 Feb 20;11:20. doi: 10.1038/s41405-025-00299-8 (PMC11842699; doi:10.1038/s41405-025-00299-8)
Supplement: Supplementary file 1 — Supplementary material [file 41405_2025_299_MOESM1_ESM.docx]

**Supplementary table 1** Mechanical properties and percentage of porosity of the sintered scaffolds

| **Group** | **Ultimate compressive strength (MPa)** | **Elastic modulus (GPa)** | **Porosity (%)** |
| --- | --- | --- | --- |
| IA | 11.1 | 0.31 | 90.3 |
| IB | 12.51 | 0.53 | 95.9 |
| IC | 9.76 | 0.47 | 96.85 |
| IIA | 11.05 | 0.47 | 91.7 |
| IIB | 4.51 | 0.41 | 99.9 |
| IIC | 6.67 | 0.46 | 98.9 |

**Supplementary table 2:** Two-way ANOVA assessing the effect of different parameters on porosity of scaffolds

| **Parameters** | **Mean Square** | **F test** | ***p* value** | **Partial Eta Squared** |
| --- | --- | --- | --- | --- |
| **Space holding agents** | 138.76 | 8.58 | 0.004* | 0.093 |
| **Mg-Zn-HA scaffolds with different HA concentrations** | 474.38 | 29.35 | <0.0001* | 0.411 |
| **Interaction** | 13.73 | 0.85 | 0.431 | 0.020 |
| **Corrected model** | 222.99 | 13.80 | <0.0001* | 0.451 |

*Statistically significant difference at *p* value ≤ 0.05, Adjusted R Squared= 0.418

**Supplementary table 3:** Pairwise comparisons between study groups regarding porosity

| **Parameters** | **Group** | **Compared to** | **Mean difference** | ***p* value** | **95% CI** |
| --- | --- | --- | --- | --- | --- |
| Group I (40% Urea) | Subgroup A | Subgroup B | 5.60 | 0.001* | 2.01, 9.19 |
|  |  | Subgroup C | 6.55 | <0.0001* | 2.96, 10.14 |
|  | Subgroup B | Subgroup C | 0.95 | 1.00 | -2.64, 4.54 |
| Group II (40% Ammonium) | Subgroup A | Subgroup B | 8.20 | <0.0001* | 4.61, 11.79 |
|  |  | Subgroup C | 7.20 | <0.0001* | 3.61, 10.79 |
|  | Subgroup B | Subgroup C | 1.00 | 1.00 | -2.59, 4.59 |
| Subgroup A | Urea | Ammonium | 1.40 | 0.343 | -1.52, 4.32 |
| Subgroup B | Urea | Ammonium | 4.00 | 0.008* | 1.08, 6.92 |
| Subgroup C | Urea | Ammonium | 2.05 | 0.166 | -0.87, 4.97 |

*Statistically significant difference at *p* value ≤ 0.05

**Supplementary table 4:** Two-way ANOVA assessing the effect of different parameters on compressive strength (MPa) of the scaffolds

| **Parameters** | **Mean Square** | **F test** | ***p* value** | **Partial Eta Squared** |
| --- | --- | --- | --- | --- |
| **Space holding agents** | 310.25 | 59.37 | <0.0001* | 0.414 |
| **Mg-Zn-HA scaffolds with different HA concentrations** | 74.23 | 14.21 | <0.0001* | 0.253 |
| **Interaction** | 120.69 | 23.10 | <0.0001* | 0.355 |
| **Corrected model** | 140.02 | 26.79 | <0.0001* | 0.615 |

*Statistically significant difference at *p* value ≤ 0.05, Adjusted R Squared= 0.592

**Supplementary table 5:** Pairwise comparisons between study groups regarding Stress (MPa)

| **Parameters** | **Group** | **Compared to** | **Mean difference** | ***p* value** | **95% CI** |
| --- | --- | --- | --- | --- | --- |
| **Group I (40% Urea)** | Subgroup A | Subgroup B | 1.41 | 0.285 | -0.63, 3.45 |
|  |  | Subgroup C | 1.34 | 0.337 | -0.70, 3.38 |
|  | Subgroup B | Subgroup C | 2.75 | 0.004* | 0.71, 4.79 |
| **Group II (40% Ammonium)** | Subgroup A | Subgroup B | 6.54 | <0.0001* | 4.50, 8.58 |
|  |  | Subgroup C | 4.38 | <0.0001* | 2.34, 6.41 |
|  | Subgroup B | Subgroup C | 2.16 | 0.034* | 0.21, 4.20 |
| **Subgroup A** | Urea | Ammonium | 0.050 | 0.952 | -1.61, 1.71 |
| **Subgroup B** | Urea | Ammonium | 8.00 | <0.0001* | 6.34, 9.66 |
| **Subgroup C** | Urea | Ammonium | 3.09 | <0.0001* | 1.43, 4.75 |

*Statistically significant difference at *p* value ≤ 0.05

**Supplementary table 6:** Two-way ANOVA assessing the effect of different parameters on the elastic modulus (GPa) of scaffolds

| **Parameters** | **Mean Square** | **F test** | ***p* value** | **Partial Eta Squared** |
| --- | --- | --- | --- | --- |
| **Space holding agents** | 0.001 | 5.50 | 0.021* | 0.061 |
| **Mg-Zn-HA scaffolds with different HA concentrations** | 0.066 | 301.19 | <0.0001* | 0.878 |
| **Interaction** | 0.156 | 708.93 | <0.0001* | 0.944 |
| **Corrected model** | 0.089 | 405.15 | <0.0001* | 0.960 |

*Statistically significant difference at p value ≤ 0.05, Adjusted R Squared= 0.958

**Supplementary table 7:** Pairwise comparisons between study groups regarding elastic modulus (GPa)

| **Parameters** | **Group** | **Compared to** | **Mean difference** | ***p* value** | **95% CI** |
| --- | --- | --- | --- | --- | --- |
| **Group I (40% Urea)** | Subgroup A | Subgroup B | 0.23 | <0.0001* | 0.21, 0.24 |
|  |  | Subgroup C | 0.16 | <0.0001* | 0.15, 0.18 |
|  | Subgroup B | Subgroup C | 0.06 | <0.0001* | 0.05, 0.08 |
| **Group II (40% Ammonium)** | Subgroup A | Subgroup B | 0.06 | <0.0001* | 0.05, 0.07 |
|  |  | Subgroup C | 0.00 | 1.00 | -0.01, 0.02 |
|  | Subgroup B | Subgroup C | 0.06 | <0.0001* | 0.04, 0.07 |
| **Subgroup A** | Urea | Ammonium | 0.16 | <0.0001* | 0.15, 0.17 |
| **Subgroup B** | Urea | Ammonium | 0.13 | <0.0001* | 0.12, 0.14 |
| **Subgroup C** | Urea | Ammonium | 0.01 | 0.135 | -0.003, 0.02 |

*Statistically significant difference at *p* value ≤ 0.05

**Supplementary table 8a:** Pairwise comparisons between study groups regarding degradation rate for spacers and different hydroxyapatite concentrations

| **Parameters** | **Group** | **Compared to** | **Mean difference** | ***p* value** | **95% CI** |
| --- | --- | --- | --- | --- | --- |
| **Group I (40% Urea)** | Subgroup A | Subgroup B | 0.26 | <0.0001* | 0.23, 0.29 |
|  |  | Subgroup C | 0.19 | <0.0001* | 0.15, 0.22 |
|  | Subgroup B | Subgroup C | 0.07 | <0.0001* | 0.04, 0.10 |
| **Group II (40% Ammonium)** | Subgroup A | Subgroup B | 0.22 | <0.0001* | 0.19, 0.25 |
|  |  | Subgroup C | 0.04 | 0.011* | 0.01, 0.07 |
|  | Subgroup B | Subgroup C | 0.18 | <0.0001* | 0.15, 0.21 |
| **Subgroup A** | Urea | Ammonium | 0.07 | <0.0001* | 0.04, 0.09 |
| **Subgroup B** | Urea | Ammonium | 0.41 | <0.0001* | 0.39, 0.44 |
| **Subgroup C** | Urea | Ammonium | 0.16 | <0.0001* | 0.13, 0.19 |

*Statistically significant difference at *p* value ≤ 0.05

**Supplementary table 8b:** Pairwise comparisons between study groups regarding degradation rate for time factor

| **Group** | **Time point** | **Compared to** | **Group I**  **(40% Urea)** | | | **Group II**  **(40% Ammonium)** | | |
| --- | --- | --- | --- | --- | --- | --- | --- | --- |
|  |  |  | **Mean difference** | ***p* value** | **95% CI** | **Mean difference** | ***p* value** | **95% CI** |
| Subgroup A | 7 days | 14 days | 0.10 | 0.067 | -0.004, 0.20 | 0.40 | <0.0001* | 0.29, 0.50 |
|  |  | 28 days | 0.41 | <0.0001* | 0.32, 0.50 | 0.60 | <0.0001* | 0.51, 0.69 |
|  |  | 35 days | 0.52 | <0.0001* | 0.44, 0.61 | 0.60 | <0.0001* | 0.51, 0.69 |
|  |  | 42 days | 0.59 | <0.0001* | 0.51, 0.67 | 0.60 | <0.0001* | 0.52, 0.68 |
| Subgroup B | 7 days | 14 days | 0.30 | <0.0001* | 0.20, 0.40 | 0.20 | <0.0001* | 0.10, 0.30 |
|  |  | 28 days | 0.40 | <0.0001* | 0.31, 0.49 | 0.50 | <0.0001* | 0.41, 0.59 |
|  |  | 35 days | 0.48 | <0.0001* | 0.40, 0.57 | 0.70 | <0.0001* | 0.61, 0.79 |
|  |  | 42 days | 0.48 | <0.0001* | 0.40, 0.56 | 0.70 | <0.0001* | 0.61, 0.78 |
| Subgroup C | 7 days | 14 days | 0.30 | <0.0001* | 0.20, 0.40 | 0.30 | <0.0001* | 0.20, 0.40 |
|  |  | 28 days | 0.48 | <0.0001* | 0.39, 0.57 | 0.50 | <0.0001* | 0.41, 0.59 |
|  |  | 35 days | 0.51 | <0.0001* | 0.43, 0.60 | 0.60 | <0.0001* | 0.51, 0.69 |
|  |  | 42 days | 0.51 | <0.0001* | 0.43, 0.59 | 0.60 | <0.0001* | 0.52, 0.68 |

*Statistically significant difference at *p* value ≤ 0.05

**Supplementary table 9a:** Pairwise comparisons between study groups regarding Mg^2+^ ion concentration for spacers and different hydroxyapatite concentrations

| **Parameters** | **Group** | **Compared to** | **Mean difference** | ***p* value** | **95% CI** |
| --- | --- | --- | --- | --- | --- |
| Group I (40% Urea) | Subgroup A | Subgroup B | 0.54 | <0.0001 | 0.50, 0.57 |
|  |  | Subgroup C | 0.41 | <0.0001 | 0.37, 0.44 |
|  | Subgroup B | Subgroup C | 0.13 | <0.0001 | 0.10, 0.17 |
| Group II (40% Ammonium) | Subgroup A | Subgroup B | 0.30 | <0.0001 | 0.26, 0.33 |
|  |  | Subgroup C | 0.18 | <0.0001 | 0.15, 0.22 |
|  | Subgroup B | Subgroup C | 0.11 | <0.0001 | 0.08, 0.15 |
| Subgroup A | Urea | Ammonium | 0.35 | <0.0001 | 0.32, 0.38 |
| Subgroup B | Urea | Ammonium | 0.49 | <0.0001 | 0.46, 0.52 |
| Subgroup C | Urea | Ammonium | 0.24 | <0.0001 | 0.21, 0.27 |

*Statistically significant difference at *p* value ≤ 0.05

**Supplementary table 9b:** Pairwise comparisons between study groups regarding Mg^2+^ concentration released during degradation for time factor

| **Group** | **Time point** | **Compared to** | **Group I**  **(40% Urea)** | | | **Group II**  **(40% Ammonium)** | | |
| --- | --- | --- | --- | --- | --- | --- | --- | --- |
|  |  |  | **Mean difference** | ***p* value** | **95% CI** | **Mean difference** | ***p* value** | **95% CI** |
| Subgroup A | 7 days | 14 days | 0.10 | 0.031* | 0.01, 0.19 | 0.38 | <0.0001* | 0.29, 0.47 |
|  |  | 28 days | 0.40 | <0.0001* | 0.31, 0.49 | 0.58 | <0.0001* | 0.49, 0.67 |
|  |  | 35 days | 0.50 | <0.0001* | 0.42, 0.58 | 0.65 | <0.0001* | 0.57, 0.74 |
|  |  | 42 days | 0.57 | <0.0001* | 0.47, 0.66 | 0.70 | <0.0001* | 0.61, 0.79 |
| Subgroup B | 7 days | 14 days | 0.24 | <0.0001* | 0.15, 0.33 | 0.20 | <0.0001* | 0.10, 0.29 |
|  |  | 28 days | 0.33 | <0.0001* | 0.24, 0.42 | 0.49 | <0.0001* | 0.40, 0.58 |
|  |  | 35 days | 0.41 | <0.0001* | 0.33, 0.50 | 0.67 | <0.0001* | 0.58, 0.75 |
|  |  | 42 days | 0.43 | <0.0001* | 0.34, 0.52 | 0.70 | <0.0001* | 0.61, 0.79 |
| Subgroup C | 7 days | 14 days | 0.30 | <0.0001* | 0.21, 0.39 | 0.19 | <0.0001* | 0.10, 0.29 |
|  |  | 28 days | 0.50 | <0.0001* | 0.41, 0.59 | 0.50 | <0.0001* | 0.41, 0.59 |
|  |  | 35 days | 0.58 | <0.0001* | 0.50, 0.67 | 0.60 | <0.0001* | 0.52, 0.69 |
|  |  | 42 days | 0.62 | <0.0001* | 0.53, 0.71 | 0.60 | <0.0001* | 0.51, 0.69 |

*Statistically significant difference at *p* value ≤ 0.05
